# Expression and Characterization of *Coprothermobacter proteolyticus* Alkaline Serine Protease

**DOI:** 10.1155/2013/396156

**Published:** 2013-12-25

**Authors:** Tanveer Majeed, Romana Tabassum, William J. Orts, Charles C. Lee

**Affiliations:** ^1^National Institute for Biotechnology and Genetic Engineering (NIBGE), P.O. Box 577, Jhang Road, Faisalabad 38000, Pakistan; ^2^Pakistan Institute of Engineering and Applied Science (PIEAS), Islamabad 44000, Pakistan; ^3^USDA-ARS, Bioproduct Chemistry and Engineering Research Unit, 800 Buchanan Street, Albany, CA 94710, USA

## Abstract

A putative protease gene (*aprE*) from the thermophilic bacterium *Coprothermobacter proteolyticus* was cloned and expressed in *Bacillus subtilis*. The enzyme was determined to be a serine protease based on inhibition by PMSF. Biochemical characterization demonstrated that the enzyme had optimal activity under alkaline conditions (pH 8–10). In addition, the enzyme had an elevated optimum temperature (60°C). The protease was also stable in the presence of many surfactants and oxidant. Thus, the *C. proteolyticus* protease has potential applications in industries such as the detergent market.

## 1. Introduction

Proteases (E.C. 3.4.21-25 and 99) are hydrolytic enzymes that degrade proteins into constituent peptides and amino acids [1]. These enzymes are commonly classified based on their functional group (aspartic, cysteine, metallo, or serine) and pH optima (acidic, neutral, or alkaline). Proteases are extremely important commercial enzymes and comprise 60% of the total market. These enzymes have wide industrial applications spanning detergents, food, leather, pharmaceuticals, and bioremediation [2]. The detergent industry is the single largest consumer of proteases which are used to remove proteinaceous stains [3]. These enzymes are required to function in the presence of diverse environmental conditions such as denaturants, metal ions, oxidants, surfactants, and elevated temperatures and pH.

Although plants and animals have proteases, the largest share of the commercially available proteases is derived from microbes. The genus *Bacillus* has been the source of the majority of alkaline serine proteases that are favored in the detergent industry because of the ease of isolation of these bacteria and the levels of enzyme activity [4–6]. Indeed, much ongoing effort is directed at the isolation of alkaline serine proteases from *Bacillus* sp. [7–11]. However, there is great value in the characterization of proteases from other bacteria to obtain enzymes with diverse activity profiles.

*Coprothermobacter proteolyticus* is an anaerobic bacterium that was isolated from a thermophilic digester fed with tannery waste and cattle manure [12, 13]. *C. proteolyticus* has an elevated optimum growth temperature of 63°C and secretes high levels of protease activity. It ferments protein more readily than casamino acids into acetate, hydrogen, and carbon dioxide. *C. proteolyticus* has recently been used in conjunction with a methanogen in the syntrophic degradation of proteinaceous substrates to produce methane [14].

We report the first expression and characterization of a recombinant *C. proteolyticus* protease. The enzyme is demonstrated to be a serine protease with an alkaline pH optimum (8–10) and functions at an elevated temperature (60°C). The protease also has the desirable property of retaining high activity in the presence of a wide variety of surfactants.

## 2. Material and Methods

### 2.1. Bacterial Strains, Plasmid, and Reagents

The strains used were *Escherichia coli* JM109 (Promega; WI, USA) and protease deficient *Bacillus subtilis* 1A751 [15] from the Bacillus Genetic Stock Center (BGSC; OH, USA). The plasmid used for *B. subtilis* expression was pDR111a (a gift from David Rudner). Bacteria were propagated in Luria-Bertani (LB) broth (*E. coli* and *B. subtilis*) or tryptose blood agar base (TBAB) (*B. subtilis*) media at 37°C. All chemicals were purchased from Sigma-Aldrich (MO, USA) unless otherwise specified.

### 2.2. Vector Construction and Gene Expression

The *C. proteolyticus aprE* was resynthesized for optimal codon usage by *E. coli* (DNA 2.0; CA, USA). The gene was then amplified by PCR and subcloned into the pDR111a vector at *Hind*III and *Sph*I restriction enzyme sites that were engineered into the 5′ and 3′ ends of the gene, respectively, to create the expression plasmid pDR111-copro-apr in *E. coli*. The plasmids pDR111a (vector control) and pDR111-copro-apr were transformed into *B. subtilis* and integrated into the bacterial chromosome using standard protocols found on the BGSC website (http://www.bgsc.org/_catalogs/Catpart4.pdf). Protein was expressed by inoculating a fresh liquid culture (OD_600_ = 0.5) with 1 mM isopropyl *β*-D-1-thiogalactopyranoside (IPTG) and growing the culture at 225 rpm at 37°C for 24 hours.

### 2.3. Protease Assay-Solid Phase

*B. subtilis* colonies transformed with either pDR111 (vector control) or pDR111-copro-apr expression plasmid were spotted onto LB agar plates containing 1% casein, 1 mM IPTG, and 100 *μ*g/mL spectinomycin antibiotic. The plates were incubated overnight at 37°C and then transferred to a higher temperature (70°C) at which the protease was active.

### 2.4. Protease Assay-Liquid

The proteolytic activity was determined using casein as the substrate. In general, 1 mL of enzyme and 1 mL of 1% casein were preincubated separately at the desired temperature for 30 min. In all cases, the pH of the enzyme and casein substrate was measured and adjusted prior to preincubation. After preincubation, the enzyme and casein were mixed and time points were collected at 0 and 20 minutes. The reactions were stopped by the addition of 3 mL of 5% trichloroacetic acid (TCA). After 10 min, the reactions were centrifuged for 5 min at 11 K ×g, and the amount of released tyrosine was measured at 275 nm. One unit of protease activity is defined as the amount of enzyme necessary to release 1 *μ*g tyrosine per minute.

#### 2.4.1. Determination of pH Optimum

The protease was pre-incubated at pH range 5–12 at 60°C for 30 min. The reaction was then initiated by adding an equal volume of 1% casein at the same pH as the enzyme. Time points were collected at 0 and 20 min, and the reactions were stopped with TCA and assayed at 275 nm as described above. 50 mM sodium succinate buffer (pH 5-6) and 50 mM sodium phosphate (pH 7.0–12.0) were used for the experiments.

#### 2.4.2. Determination of Optimum Temperature

The enzyme and casein substrate were pre-incubated separately at different temperatures (50–90°C) at pH 9 for 30 min. Then, the enzyme and substrate were combined, and the reaction proceeded at the pre-incubation temperature. Time points were collected at 0 and 20 min, and the level of proteolysis was assayed as described above.

#### 2.4.3. Determination of Thermal Stability

The protease was heated at different temperatures (50–90°C) at pH 9 for 0, 30, and 60 min. After the heat challenge, the enzyme was pre-incubated for 30 min at 60°C, and then the reaction was initiated by the addition of 1% casein substrate. The reaction proceeded at 60°C and was assayed as described above.

#### 2.4.4. Determination of Additives Effect

Various metal cations (Ca^+2^, calcium chloride; Co^+2^, cobalt chloride; Fe^+2^, ferrous chloride; Mg^+2^, magnesium chloride; Mn^+2^, manganese sulfate; Ni^+2^, nickel chloride; and Zn^+2^, zinc sulfate) were pre-incubated with the protease for 30 min at 60°C. Various protease inhibitors (*β*-mercaptoethanol, *β*-ME; dithiothreitol, DTT; ethylenediaminetetraacetic acid, EDTA; iodoacetate, IAA; and phenylmethylsulfonyl fluoride, PMSF) were added to the protease for 30 min at 37°C and then pre-incubated at 60°C for 30 min. Surfactants (sodium dodecyl sulfate, Triton X-100, and Tween-20) and oxidant (H_2_O_2_) were added to the protease for 60 min at 37°C and then pre-incubated at 60°C for 30 min. All additives were used at a final concentration of 5 mM. All incubations occurred at pH 9. After pre-incubations, casein was added to all the enzymes, the reactions proceeded at 60°C, and residual protease activities were assayed as described above.

## 3. Results and Discussion

### 3.1. Gene Cloning and Expression

**Figure 1:**
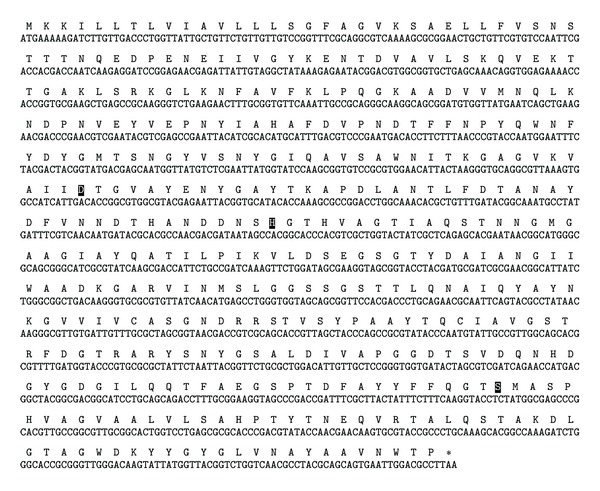
*aprE* resynthesized gene sequence and translation. Highlighted residues indicate the predicted catalytic triad of known serine proteases.

A putative protease gene (*aprE*) from the thermophilic *C. proteolyticus* DSM 5265 was chosen as the target of study. The gene sequence (NCBI NC_011295) was resynthesized to conduct codon optimization for *E. coli* expression (Figure 1). The amino-acid sequence of the protein has the predicted conserved catalytic triad residues of known serine proteases [16]. When the sequence was compared to the NCBI database by BLAST analysis, a peptidase from *Caldisericum exile* AZM16c01 (NCBI YP_005473527.1) was the closest match with 74% identity [17].

**Figure 2:**
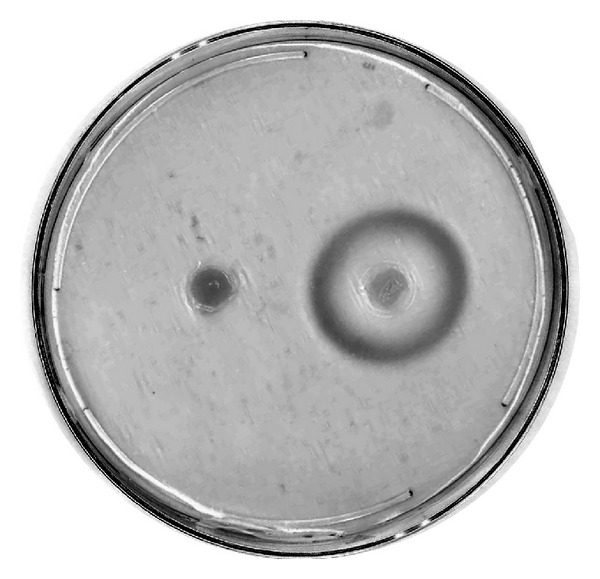
*B. subtilis* protease expression. Colonies transformed with either vector control (left) or *aprE* expression construct (right) were spotted onto LB agar plates containing 1% casein and 1 mM IPTG.

The *aprE* gene was cloned into an *E. coli* expression vector (pET29b+; Novagen, WI, USA). This plasmid was either transformed into an *E. coli* expression strain (BL21(DE3) pLysE; Novagen) or used as a template in an *in vitro* transcription/translation reaction (*E. coli* T7 S30 Extract System; Promega). Only the *in vitro *transcription/translation reaction yielded recombinant enzyme, and no protease activity was detected (data not shown). Therefore, the gene was subcloned into a *B. subtilis* expression vector downstream of an IPTG-inducible promoter. The expression plasmid was transformed into a protease-deficient *B. subtilis* strain, and an active recombinant enzyme was secreted from the cells (Figure 2). *B. subtilis* transformed with the expression vector produced high levels of enzyme activity in liquid culture medium 24 hours after IPTG induction, whereas control *B. subtilis* carrying only vector DNA resulted in no significant protease activity (data not shown).

### 3.2. pH and Temperature Optima

**Figure 3:**
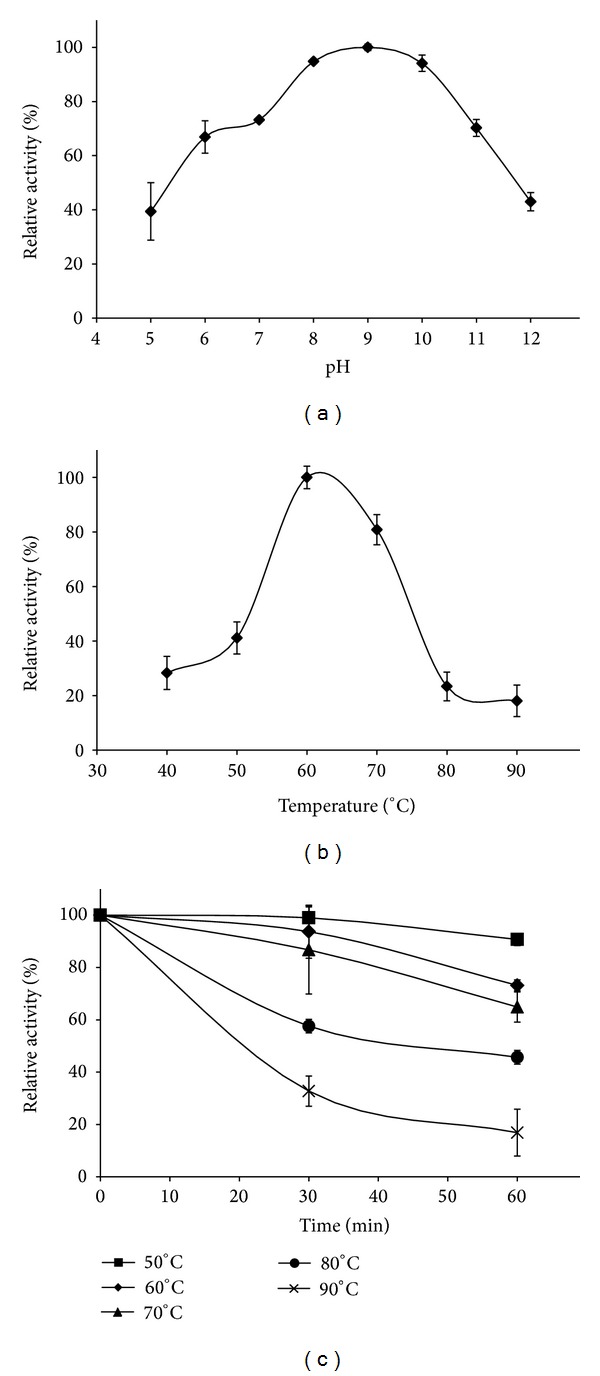
Effect of pH and temperature on activity. (a) Activity of protease at various pH and 60°C. (b) Activity of protease at various temperatures and pH 9. (c) Activity of protease after heat treatment. Residual activity was assayed at 60°C and pH 9.

The activity of the recombinant protease was tested under a variety of pH values. The enzyme had the highest activity from pH 8 to pH 10 (Figure 3(a)). At pH 7 and pH 11, the protease retained 70% of the activity. The temperature optimum was determined to be 60°C (Figure 3(b)). This is consistent with the optimal growth temperature (63°C) of the source organism. When the reaction was conducted at 70°C, the activity decreased to 80%. Under optimal conditions (pH 9 and 60°C), there was 66 U/mL of activity.

The thermostability of the protease was tested at various temperatures and times (Figure 3(c)). At 50°C, there is a slight drop in activity after 60 min. At 60°C and 70°C, there is a larger decrease to approximately 70% after 60 min. At 80°C, half the activity is lost at 60 min. Finally, at 90°C, only 30% of the activity remains after 30 min.

### 3.3. Effect of Additives on Protease Activity

**Figure 4:**
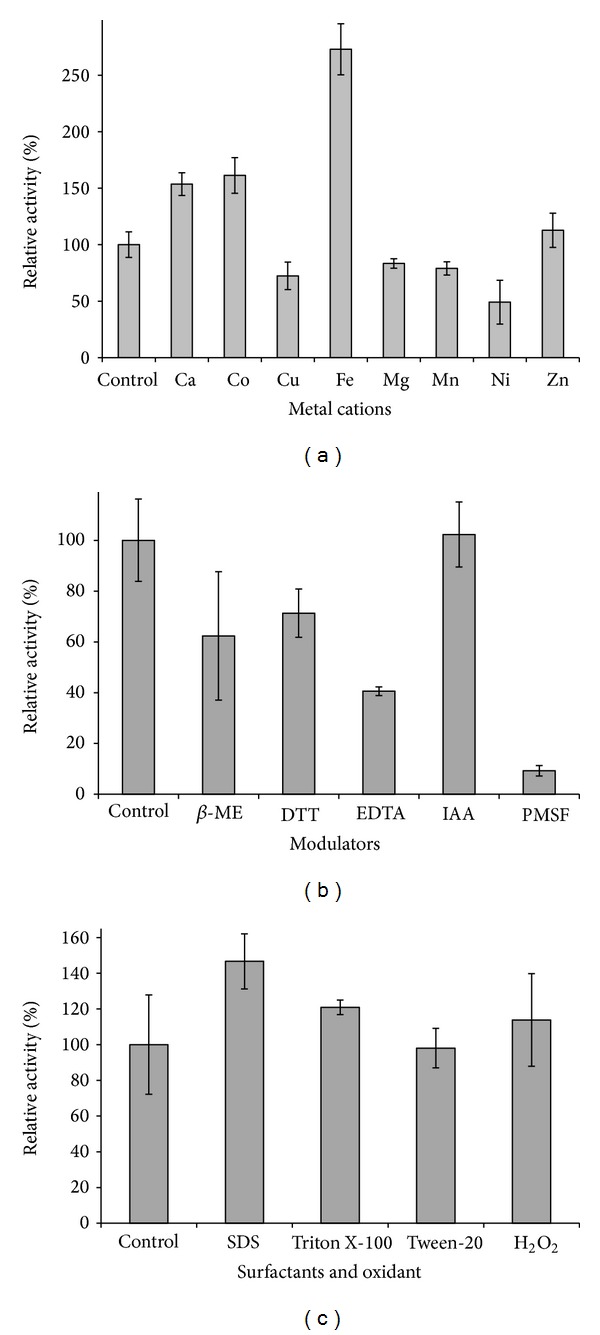
Effect of additives on activity. (a) Activity of protease after incubation with various metal cations. (b) Activity of protease after incubation with various inhibitors. (c) Activity of protease after incubation with various surfactants and oxidants. All residual activities were assayed at 60°C and pH 9. Control reactions contain no additives.

The effects of numerous divalent cations on protease activity were tested (Figure 4(a)). Fe^+2^ greatly stimulated the protease activity to 248% of the unsupplemented enzyme level. Ca^+2^ and Co^+2^ both stimulated enzyme activity to approximately 150%. Cu^+2^, Mg^+2^, Mn^+2^, and Ni^+2^ decreased enzyme activity to 49–83%, while Zn^+2^ had little effect.

Addition of PMSF dramatically reduced enzyme activity, thus supporting the identity of this enzyme as a serine protease (Figure 4(b)). EDTA reduced activity to 40% which could be a reflection of the cation influence on protease activity seen in Figure 4(a). The *β*-ME and DTT reducing agents both induced moderate inhibition, while IAA had no effect.

The protease was highly stable in a variety of surfactants and oxidant (Figure 4(c)). All these additives resulted in either no effect or increased activity. SDS induced the greatest stimulation at 146% relative to unsupplemented enzyme.

## 4. Conclusion

This is the first report of the biochemical characterization of a recombinant protease from the thermophilic *C. proteolyticus*. The enzyme was demonstrated to be an alkaline serine protease that was active at elevated temperatures and resistant to many surfactants, thus indicating potential utility of this enzyme in detergent applications. In addition, the new protein sequence of this enzyme will be of great value in the continued efforts to develop protease activity improvements [18–20]. One of the key strategies of enzyme engineering is directed evolution through DNA shuffling between different family members [21, 22]. The availability of more unique amino acid sequences from proteases whose activities have been experimentally validated is critical to such projects.
